# Closing the door to false memory: the effects of levels-of-processing and stimulus type on the rejection of perceptually vs. semantically dissimilar distractors

**DOI:** 10.1007/s00426-021-01544-z

**Published:** 2021-06-10

**Authors:** Marek Nieznański, Michał Obidziński

**Affiliations:** Institute of Psychology, Cardinal Stefan Wyszyński University in Warsaw, ul. Wóycickiego 1/3 bud. 14, 01-938 Warsaw, Poland

## Abstract

False recognition memory for nonstudied items that share features with targets can be reduced by retrieval monitoring mechanisms. The recall-to-reject process, for example, involves the recollection of information about studied items that disqualifies inconsistent test probes. Monitoring for specific features during retrieval may be enhanced by an encoding orientation that is recapitulated during retrieval. In two experiments, we used concrete words or door scenes as materials and manipulated the level of processing at study and the type of distractors presented at test. We showed that for the verbal material, semantic level of processing at study results in an effective rejection of semantically inconsistent distractors. However, for the pictorial material, the perceptual level of processing leads to an effective rejection of perceptually inconsistent distractors. For targets, the effect of levels of processing was observed for words but not for pictures. The results suggest that retrieval monitoring mechanisms depend on interactions between encoding orientation, study materials, and differentiating features of distractors.

## Introduction

Incidents of false memories depend on two classes of processes: error-inflating processes that are generally based on familiarity (activation) increased by the shared attributes of targets and lures, and error-editing processes that overall depend on the recollection (monitoring) of features that are distinctive (e.g., Arndt & Gould, 2006). The recollection of study details can be used to avoid false recognition through such decision mechanisms as disqualifying monitoring or diagnostic monitoring (Gallo, 2006). The first occurs when the remembering of one event logically excludes another event as being presented during the study, while the second mechanism is based on the failure to recollect the expected details (cf. Nieznański et al., 2018). For example, in a converging associates memory task, a disqualifying monitoring process can lead to rejection of a related lure because one recalls identifying it as nonstudied during study, whereas a diagnostic monitoring process can result in rejecting an unrelated lure because it does not fit the gist of studied items (Gallo, 2006, p. 204). When the distinctiveness of the study material is enhanced, retrieval monitoring becomes more effective since subjects can make use of the unique features and qualitative differences between targets and lures (Gray & Gallo, 2015). One of the manipulations that was used to influence such recollective distinctiveness was a deep vs. shallow level of processing (Gallo et al., 2008). Our aim was to investigate the effects of levels of processing on disqualifying monitoring depending on the kind of study material (concrete words vs. door scenes). In two experiments, we crossed encoding tasks orienting subjects’ attention toward semantic vs. perceptual features with semantic vs. perceptual features that may be used to exclude lures at retrieval.

In false memory literature, “recall-to-reject” processes are described that involve the recollection of information which eliminates the recognition probe as having occurred (e.g., Gallo, 2004; Gallo et al., 2006, 2007). In other words, these processes facilitate the rejection of test probes that are similar to targets by detecting their mismatch on some of the features (e.g., Carneiro et al., 2012; Rotello et al., 2000). For example, a subject may recollect the colours of the studied targets that are different from the colours of the recognition probe, thus, rejecting it as a lure. However, there are at least two necessary conditions for the effective use of such a strategy. The first involves recalling the entire range of features presented at study that are relevant for the testing probes. The second is adopting a proper retrieval orientation that enables the monitoring of distinctive information at retrieval.

The classic transfer appropriate processing framework (Morris et al., 1977) suggests that performance on a memory task is enhanced by increases in the overlap between the processes carried out during encoding and those carried out during test (e.g., Nieznański, 2014). More recent research posits that people attempt to take advantage of transfer appropriate processing by recapitulating the operations performed at the time of encoding during retrieval (e.g., Alban & Kelley, 2012; Zawadzka et al., 2017). Therefore, subjects search memory differently when their task is to recognize items that were encoded with a semantic task than when the task is to recognize perceptually encoded items (Kantner & Lindsay, 2013). Jacoby and colleagues (e.g., Halamish et al., 2012; Jacoby et al., 2005) use the notion of *source-constrained retrieval* for a kind of early selection in which the retrieval processing is constrained in a way that recapitulates study processing (cf. Alban & Kelly, 2012; Danckert et al., 2011; Marsh et al., 2009). A related concept of *retrieval orientation* was proposed by Rugg and colleagues (e.g., Morcom & Rugg, 2012; Rugg & Wilding, 2000) for a goal-directed strategy adopted at retrieval. They demonstrated—using brain imaging and evoked potentials—that new items on a recognition memory test are processed in a way that depends on how the targets were encoded at study (cf. Kantner & Lindsay, 2013; Zawadzka et al., 2017).

### Levels-of-processing effect with verbal vs. pictorial material

The levels-of-processing (LoP) effect refers to the well-known impact of orienting tasks during study on subsequent memory test performance. A hierarchy of qualitatively different levels of analysis can be defined, starting with a sensory (shallow) analysis of the perceptual properties of the to-be-remembered items, and proceeding towards a more elaborate (deep) processing of meaning and semantic associations of the items (e.g., Craik, 2002; Craik & Lockhart, 1972). As shown in numerous studies, semantically encoded items are typically better remembered than perceptually encoded stimuli (e.g., Craik & Tulving, 1975). Such positive LoP effects were usually studied and demonstrated with verbal material. However, a relatively small number of studies have used pictorial material, yielding considerably mixed results (Baddeley & Hitch, 2017; Nieznański, 2020). In some studies, particularly those using pictures from a single broad category (e.g., faces or door scenes), semantic processing led to superior memory performance (Baddeley & Hitch, 2017; Bower & Karlin, 1974; Konstantinou & Gardiner, 2005). But in other studies using more distinctive pictorial material, reversed LoP effects have been reported. For example, in the Intraub and Nicklos (1985) study, questions orienting participants towards the visual characteristics of objects led to a better recall than semantic questions. In our recent study (Nieznański, 2020) using the conjoint recognition paradigm, we also found significant negative LoP effects for pictures. Process-level analyses of the components involved in memory performance that we conducted from the perspective of the dual-recollection theory (Brainerd et al., 2014, 2015) demonstrated a significant enhancement of pictures’ context recollection and null effects for target recollection in the perceptual encoding condition in comparison with the semantic encoding condition. In contrast, for verbal material, both context and target recollection were enhanced in the semantic condition. Following the sensory-semantic model (Nelson & Reed, 1976; Nelson et al., 1976, 1977), we can assume that picture recollection may benefit from perceptual encoding due to the greater physical distinctiveness of pictures in comparison with words (Intraub & Nicklos, 1985). However, restricting picture diversity by using stimuli similar in appearance might eliminate this advantage and render semantic features relatively more effective in differentiating the targets from the distractors (cf. Baddeley & Hitch, 2017).

Modest but consistently positive LoP effects were recently reported by Baddeley and Hitch (2017) in a series of experiments using sets of visual stimuli taken from single categories such as doors, clocks or mobile phones. When comparing recognition memory for pictures and words, in one of the experiments, Baddeley and Hitch used lists of door scenes, half of which were predominantly brown, and lists of concrete words printed in various font colours, half of which contained a majority of brown letters. The stimuli were processed either deeply, where the participants judged if they found each item pleasant, or shallowly, in terms of assessing whether the stimulus was predominantly brown. At a four-alternative forced-choice recognition test, three similar distractor items were presented along with the target, but the type of the target–distractor similarity was not systematically manipulated. Deep encoding resulted in better memory in the case of both doors and words, but the effect was markedly smaller for pictorial than for verbal materials. In subsequent experiments, modest LoP effects were confirmed with various pictorial stimuli, whereas for verbal materials, the effects varied much more depending on the available features associated with the verbal material. Such features, according to Baddeley and Hitch, are “offered” by stimuli to be elaborated and may potentially enhance target encoding and their differentiation among distractors. The more diagnostic features are processed, the greater the probability of successful recognition. Baddeley and Hitch proposed the concept of *affordances* taken from James J. Gibson’s (1977) classical theory of perception as a suitable term for expressing the relationship between the subject and the to-be-remembered materials. From this perspective, a word affords a relatively impoverished perceptual stimulus, but one that can be referred to a rich and complex network of lexical associations. In contrast, a picture of a domestic door affords a broad range of visual features that might enhance stimulus familiarity at retrieval, but are relatively useless as diagnostic features when presented among similar domestic doors as distractors. Taking into account the differences between the perceptual and semantic features in their potential impact on recognition memory, Baddeley and Hitch proposed a distinction between *perceptual* and *semantic affordances*. In the current study, we further investigate the differences in the consequences of perceptual and semantic feature processing, this time for false memory. We systematically manipulate the type of features in which the distractors share or differ in relation to the targets, rendering the presence (or absence) of certain features useful for retrieval monitoring.

### Overview of the experiments

The aim of our research is to demonstrate that retrieval monitoring depends on the interaction between level of processing, materials, and lure type (cf. Chan et al., 2005). Across experiments we compare the usefulness of perceptual and semantic affordances (Baddeley & Hitch, 2017) in retrieval monitoring for words vs. pictures. We hypothesize that subjects monitor their memory for specific features that were distinguished at study by orienting tasks. In particular, we assume that subjects who focus their attention on the colours of the target at study attempt to recapitulate this orientation at retrieval, in consequence, the targets and lure items are monitored for containing appropriate colours. Conversely, subjects who focus on the semantic categories of targets at study disqualify the lures belonging to new categories at test. Moreover, such retrieval orientations should be more successful the more distinctive are the features of targets. Previous research (e.g., Gallo et al., 2008) suggests that a deep level of processing enhances the recollective distinctiveness for words. However, it is not clear whether the same effect will be present for pictures. On the one hand, the research of Baddeley and Hitch (2017) suggested that the distinctiveness of such pictorial material as door scenes will benefit from semantic processing in comparison with the perceptual encoding task (at least when nondistinctive distractors are used at test). On the other hand, our recent research (Nieznański, 2020) has indicated that context recollection for pictures is enhanced by perceptual processing. Hence, it is probable that the colour orienting condition will result in more effective colour recollection and, in consequence, better disqualification of colour-inconsistent lures.

In Experiments 1 and 2, we used similar materials to Baddeley and Hitch (2017), that is, lists of concrete words printed in coloured fonts (Experiment 1) and lists of door scenes (Experiment 2) encoded either under a semantic or a colour orienting task. At test, in contrast to the Baddeley and Hitch studies, we manipulated the kind of lures that were presented, that is, we used “colour lures” containing colours consistent with the colours presented at study but belonging to inconsistent categories, “category lures” belonging to consistent categories but containing inconsistent colours, and “critical lures” that were consistent both in category and colour with the study items. According to the global matching models of recognition memory (e.g., Arndt, 2015), false recognition is a function of the match between a lure used as a memory probe during test and the memory traces of related targets. Therefore, memory performance in our experiments will depend both on the error-inflating processes based on lure consistency, and the error-editing processes based on lure inconsistency with the encoded traces.

In both experiments, apart from the standard analyses of hit rates, false alarm rates, and mean confidence of responses, we used two alternative measurement models, namely the signal detection (SDT) model, and the two-high-threshold model (2HT) for recognition memory. Such twofold modelling analysis was motivated by a still indecisive debate in literature as to whether recognition performance is better described as based on a continuous memory process (SDT) or discrete states (2HT) (e.g., Bröder & Schütz, 2009; Dube et al., 2012; Juola et al., 2019; Malejka & Bröder, 2019). Both measurement models enable some specific interpretations of the results. For example, on the one hand, Huff and Bodner (2013) have recommended SDT analyses as a way to disentangle encoding (generally, error-inflating) from retrieval (generally, error-editing) influences. The latter would rather affect the response criterion parameter, while the former is expected to influence the memory sensitivity parameter of the SDT model (cf. Nieznański et al., 2018). On the other hand, the 2HT model introduces a parameter representing high-confidence “no” responses for lures detected as distractors, which may be interpreted as a manifestation of the recall-to-reject process (Rotello et al., 2000). This way we can compare effectiveness of this monitoring mechanism between lure types depending on the encoding condition.

## Experiment 1

### Methods

#### Participants

The participants were 53 undergraduates who received course extra credits for volunteering. Their mean age was 19.9 years (SD = 0.90); 11 were men. Each participant was assigned to one of two conditions differing by the encoding instructions: colour naming (*N* = 27) and category naming (*N* = 26). The numbers of participants per group were similar to the numbers of participants in the Baddeley and Hitch (2017) Experiments 1 (*N* = 20) and 2 (*N* = 24), which used similar materials and conditions. A sensitivity analysis using G*Power 3.1 (Faul et al., 2007) revealed that, assuming a power of 0.80, with our sample size (*N* = 53), the experiment is sufficiently sensitive to detect a small-to-medium effect size of *f* = 0.18, for ANOVA with repeated measures and within–between interaction.

### Materials

The material comprised a list of 78 nouns (mean length 6.4 letters, ranging from 3 to 11 letters) belonging to six different semantic categories and containing a majority of letters in one of six font colours. These words were assigned to the following sets: (a) 45 targets: 15 names of animals, 15 clothes, and 15 fruits; for each of these semantic categories a third was printed predominantly in red, a third in green, and a third in brown font colour; (b) nine critical lures: words belonging to the same sematic categories and containing a majority of letters in the same colours as the targets; (c) nine category lures: words belonging to the same semantic categories as the targets but differing in the predominant font colour (grey, blue or yellow); (d) nine colour lures: words with a majority of red, green or brown letters (as targets) but differing from the targets in the semantic category (furniture, tools or musical instruments); and (e) six study items similar in category and colour to the targets serving as primacy and recency buffers. All the words were presented in 60-point Times New Roman font. The first letter of each word was in its predominant font colour. For shorter words, all the letters except one were presented in the predominant colour; for longer words, two or three letters were presented in different nondominant colours. When the majority of letters were presented in red, green or brown, the colours of the remaining letters were chosen at random from grey, blue or yellow colours, and vice versa. The background screen was black.

#### Procedure

**Fig. 1:**
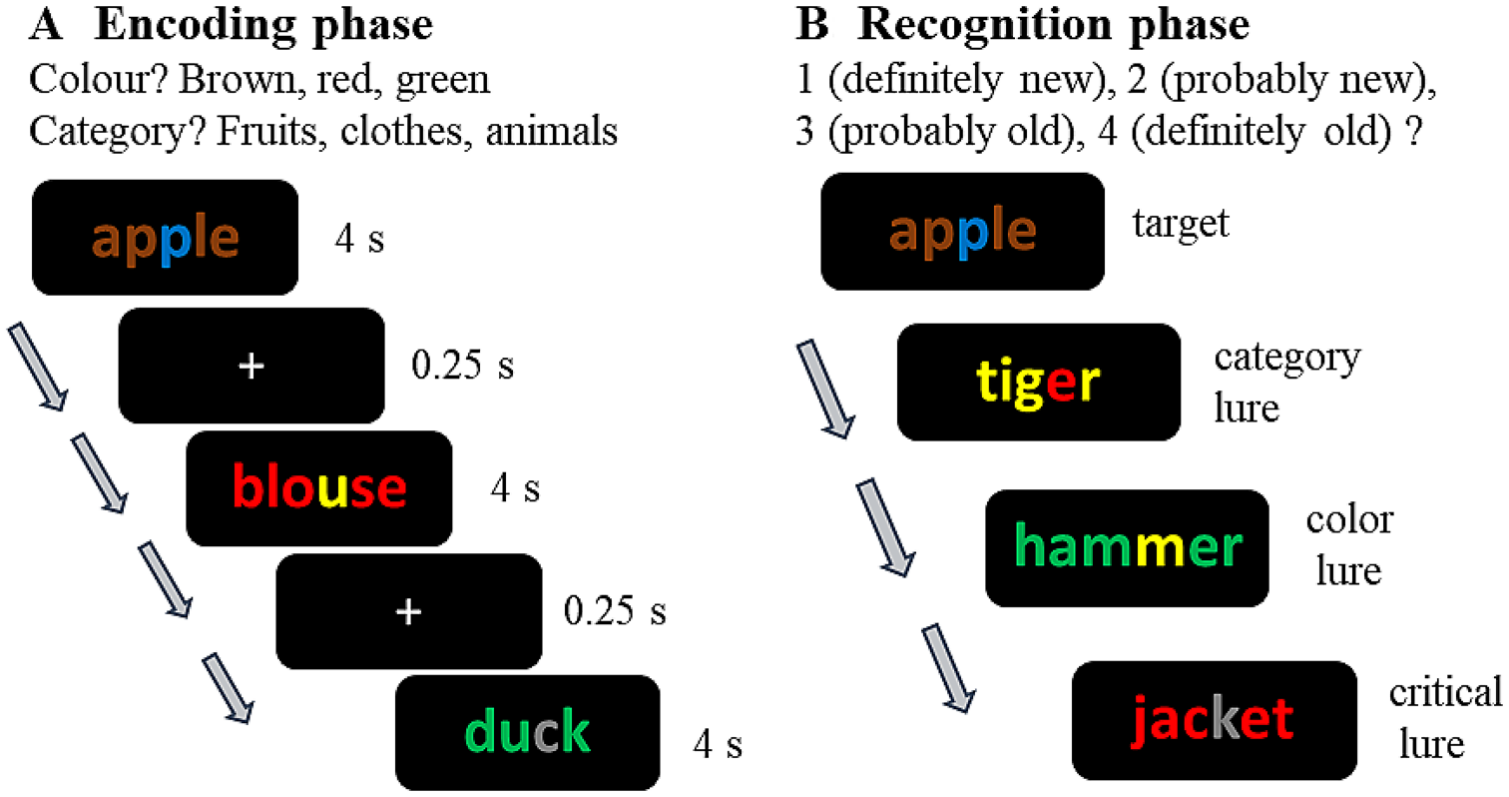
Diagram of the procedure in Experiment 1. During the encoding phase, participants were asked to judge either the dominant colour of letters or the category to which the word belongs, this orienting task was manipulated between-subjects. During the test phase, the participants recognized whether the word was presented or not using a 4-point confidence scale. Targets were mixed with three kinds of lure items: category lures, colour lures, and critical lures

The procedure is schematically depicted in Fig. 1. At study, the participants were instructed to try to memorize all the presented words and to answer the orienting question using a keyboard. In the category condition, the participants were asked to press one of three keys that corresponded to the animals, clothes or fruits categories. In the colour condition, they were asked to indicate whether most letters of the presented word are brown, green or red.

The 45 target words (3 colours × 3 categories × 5 exemplars) were presented at study in a random order at a rate of 4 s with an interstimulus interval of 250 ms. Three words were added as buffers at the beginning and another three at the end of the study list. All the words were displayed in the centre of the computer screen. The response options were prompted in a white frame below the target word.

At test, 27 targets (3 colours × 3 categories × 3 exemplars) mixed with 27 distractors (9 critical lures, 9 category lures, and 9 colour lures) were presented in a random order. The participants were asked to recognize items using a 4-point confidence scale: 1 (definitely new), 2 (probably new), 3 (probably old), 4 (definitely old). The response options were displayed in a white frame at the bottom of the slide. The test trials were participant-paced with the next trial appearing immediately after a response.

The participants were examined at individual workstations in the University Lab. The presentation of the stimuli and the response recording were controlled using the E-Prime program 2.0 (Psychology Software Tools, Pittsburgh, PA).

### Data analysis

*Analysis of variance (ANOVA)* An α level of 0.05 was used for all statistical tests. For repeated measures ANOVA, whenever the assumption of sphericity was not met, as indicated by Mauchly’s test, we reported Greenhouse–Geisser corrected degrees of freedom. For a part of our dependent variables, we found that the assumption of distribution normality was not met; however, we decided to conduct parametric ANOVAs, taking into account the suggestions in literature about *F*-test robustness to violations of normality (e.g., Blanca et al., 2017).

*Signal-detection measurement model* Calculations of estimates of signal-detection parameters were performed using *SDT Assistant* software (Hautus, 2014). It provides maximum likelihood estimates of parameters using a quadratic convergence procedure. We assumed the unequal-variance normal model and computed *d*_*a*_ as the memory sensitivity parameter (Simpson & Fitter, 1973) and *x*_*c*_ as the response criterion location; we reported only the placement of the middle criterion, which divides the decision axis into positive (“definitely old” and “probably old”) and negative responses (“definitely new” and “probably new”). Because of a low number of trials collected from each participant, we calculated the signal-detection model parameters from pooled data (Hautus, 1997). The hypotheses about the differences between the parameters were tested using the *z* statistic in the manner recommended by Wickens (2002, Ch. 11.4).

**Fig. 2:**
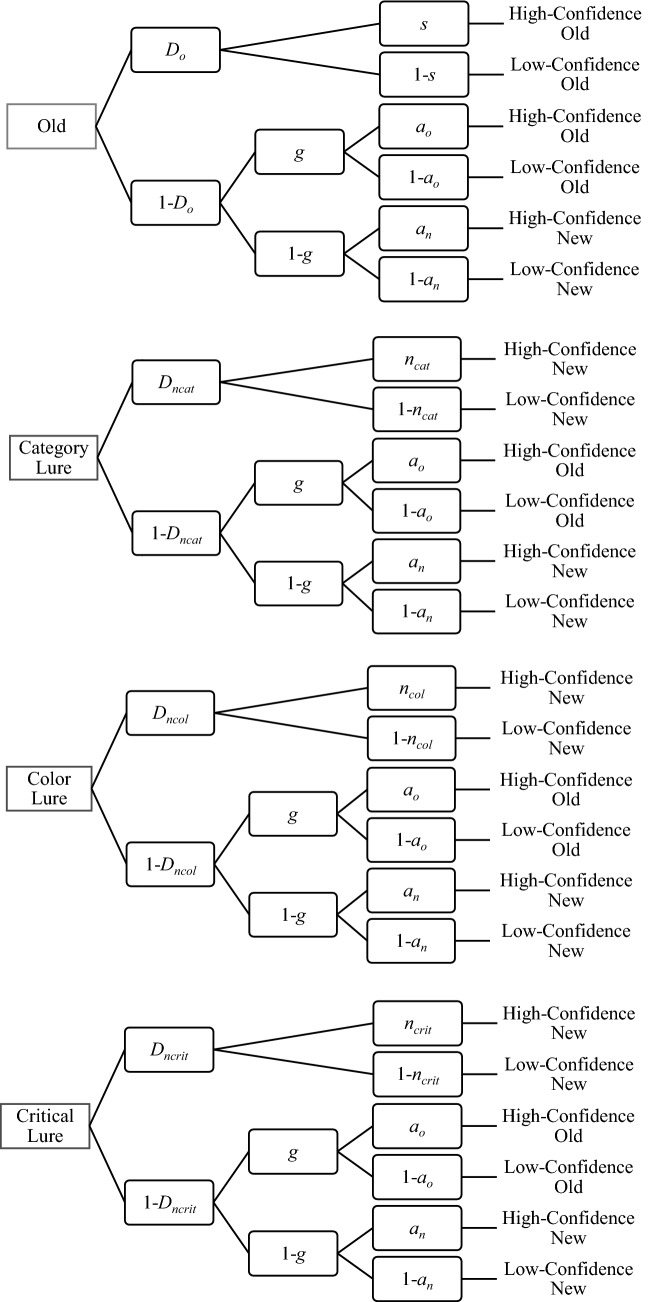
Two-high threshold multinomial processing tree model

*Multinomial processing tree model* The two high-threshold model (2HTM) is a discrete-state model which assumes that recognition memory is mediated by discrete ‘‘detect’’ and ‘‘guessing’’ states (Kellen et al., 2015). A graphical representation of the 2HT multinomial processing tree model used in the current research is depicted in Fig. 2. It was based on the version of 2HTM presented by Kellen et al., (2015, see Fig. 1). According to this model, parameter *D*_*o*_ represents the probability that an old item is detected, leading to a “definitely old” response (with probability *s*) or “probably old” response (with probability 1—*s*). If the old item is not detected (1—*D*_*o*_), the status of the item is guessed as old, with probability *g*, or as new (1—*g*). For items guessed as old, high or low confidence “old” responses are chosen with probability *a*_*o*_ or (1—*a*_*o*_), respectively. For undetected items guessed as new, the “definitely new” response is chosen with probability *a*_*n*_, and the “probably new” response with probability (1—*a*_*n*_). A new item presented at test is detected with probability *D*_*n*_, leading to a ‘‘new’’ response with high confidence (parameter *n*) or low confidence (1—*n*). In the current research, we assume that both the *D*_*n*_ and *n* parameters can differ depending on the kind of lure used at test. When detection of a new item fails (1—*D*_*n*_), a guessing state is entered into, which is assumed to be the same as in the case of undetected old items, and identical for all kinds of lures. The goodness of fit of the model to the empirical data was tested with the log-likelihood ratio statistic (*G*^2^), which is distributed asymptotically as a *χ*^2^ distribution. At α level of 0.05, *G*^2^(1) = 3.84 indicates a critical value. Computations were carried out with the *multiTree* computer program (Moshagen, 2010). Some of the parameter estimates (e.g., parameter *s*) were close to the upper boundary of the parameter space (i.e., near 1). In such a case, the use of bootstrap simulations is recommended to draw inferences regarding the variability of the parameter estimates (Moshagen, 2010; Singmann & Kellen, 2013).

## Results and discussion

At study, the participants almost perfectly answered the orienting questions (98.5% and 98.8%, of the responses were correct^1^ for the category and colour conditions, respectively). The raw data as well as the tables presenting the hit rates and false alarm rates across response criteria for both experiments are available at https://osf.io/st6rc/.

### Standard statistical analyses (ANOVA)

Mean proportions of acceptances of targets and lures across confidence levels are presented in the upper half of Table 1. For the hit rate (i.e., the proportion of “definitely old” and “probably old” responses to targets) as the dependent variable, one-way ANOVA examining the effect of encoding task (colour orienting task, category orienting task) revealed a significantly higher hit rate in the category condition (*M* = 0.85, SD = 0.102) than in the colour condition (*M* = 0.75, SD = 0.139), *F*(1) = 9.61, *p* < 0.01, *η*_*p*_^2^ = 0.16.

**Table 1:** Mean (SD) proportions of targets and lures classified to four confidence levels, and the mean (SD) confidence ratings of items in Experiments 1 and 2, depending on the encoding condition

Study conditions	Definitely new (1)	Probably new (2)	Probably old (3)	Definitely old (4)	Mean rating
Experiment 1					
Colour encoding					
Targets	0.089 (0.1029)	0.165 (0.1013)	0.199 (0.1320)	0.547 (0.1936)	3.204 (0.3706)
Category lures	0.568 (0.3148)	0.309 (0.2779)	0.074 (0.1022)	0.049 (0.0712)	1.605 (0.4034)
Colour lures	0.436 (0.3080)	0.370 (0.2615)	0.123 (0.1125)	0.070 (0.0982)	1.827 (0.4501)
Critical lures	0.362 (0.2679)	0.391 (0.2434)	0.140 (0.1293)	0.107 (0.1173)	1.992 (0.4052)
Category encoding					
Targets	0.044 (0.0445)	0.105 (0.0958)	0.181 (0.1351)	0.669 (0.2042)	3.476 (0.3073)
Category lures	0.547 (0.2879)	0.316 (0.2259)	0.103 (0.1214)	0.034 (0.0755)	1.624 (0.4344)
Colour lures	0.859 (0.2098)	0.098 (0.1841)	0.021 (0.0546)	0.021 (0.0546)	1.205 (0.2858)
Critical lures	0.393 (0.2577)	0.325 (0.2176)	0.162 (0.1482)	0.120 (0.1631)	2.008 (0.4796)
Experiment 2					
Colour encoding					
Targets	0.059 (0.0971)	0.105 (0.0902)	0.214 (0.1401)	0.622 (0.1979)	3.400 (0.3652)
Category lures	0.857 (0.2919)	0.062 (0.1405)	0.052 (0.1605)	0.029 (0.1029)	1.252 (0.5621)
Colour lures	0.738 (0.2978)	0.152 (0.1869)	0.081 (0.1636)	0.029 (0.0856)	1.400 (0.5516)
Critical lures	0.270 (0.1892)	0.251 (0.1533)	0.289 (0.1730)	0.190 (0.1607)	2.400 (0.4413)
Category encoding					
Targets	0.063 (0.0831)	0.105 (0.1230)	0.148 (0.1180)	0.684 (0.2382)	3.452 (0.4518)
Category lures	0.602 (0.3119)	0.222 (0.2289)	0.097 (0.1754)	0.079 (0.1290)	1.653 (0.5914)
Colour lures	0.755 (0.3368)	0.120 (0.2055)	0.069 (0.1661)	0.056 (0.1127)	1.426 (0.6368)
Critical lures	0.321 (0.2446)	0.278 (0.2262)	0.219 (0.1996)	0.182 (0.1724)	2.262 (0.5110)

For the false alarm rate (i.e., the proportion of “definitely old” and “probably old” responses to lures) a 3 (distractor type) × 2 (encoding task) mixed ANOVA was calculated, with the distractor type (critical lure, category lure, and colour lure) manipulated within-subjects, and the encoding task (colour orienting task, category orienting task) manipulated between subjects. Main effects of distractor type, *F*(2) = 22.71, *p* < 0.001, *η*_*p*_^2^ = 0.31, and an interaction effect, *F*(2) = 8.86, *p* < 0.001, *η*_*p*_^2^ = 0.15, were revealed; however, no effect of encoding condition was observed, *F*(1) = 1.56. Post hoc comparisons showed significantly more false alarms for critical lures (*M* = 0.26, SD = 0.168) than category lures (*M* = 0.13, SD = 0.134), *t*(52) = 5.68, *p*_Bonf_ < 0.001, *d* = 0.78, and the colour lures (*M* = 0.12, SD = 0.139), *t*(52) = 5.32, *p*_Bonf_ < 0.001, *d* = 0.73, but there was no difference in false alarm rate between the category lures and the colour lures, *t*(52) = 0.39. No significant differences were found in false alarm rate for the category and critical lures depending on the encoding condition; however, for the colour lures, higher rate was observed in the colour condition (*M* = 0.19, SD = 0.143) than in the category condition (*M* = 0.04, SD = 0.084), *t*(42.17) = 4.70, *p* < 0.001, *d* = 1.29.

The results concerning confidence ratings are parallel to the results described above for hit rates and false alarm rates and they are presented in the “Appendix 1”.

### Signal detection analyses

The upper part of Table 2 presents SDT parameter estimates based on data pooled over the participants. Memory sensitivity parameter *d*_*a*_ comparisons between the encoding conditions showed a better sensitivity in the category condition than in the colour encoding condition. In detail, in the category condition, memory sensitivity was better than in the colour condition when category lures, *z* = 3.14, *p* < 0.002, colour lures, *z* = 8.38, *p* < 0.001, and critical lures, *z* = 2.82, *p* < 0.005, were used for the calculations of the false alarm rates. The placement of the middle response criterion *x*_*c*_ for colour lures was significantly more conservative in the category condition than in the colour condition, *z* = 5.45, *p* < 0.001. In the case of the category lures and the critical lures, no significant differences were found in the placement of the response criteria between the encoding conditions.

**Table 2:** Signal detection parameter estimates (SE) based on data pooled over participants

Study conditions	Targets vs. category lures	Targets vs. colour lures	Targets vs. critical lures
Experiment 1			
Colour encoding			
* d*_*a*_	1.667 (0.0864)	1.422 (0.0811)	1.250 (0.0800)
* x*_*c*_	1.086 (0.0920)	0.818 (0.0833)	0.621 (0.0791)
Category encoding			
* d*_*a*_	2.063 (0.0921)	2.716 (0.1314)	1.591 (0.0904)
* x*_*c*_	1.067 (0.0933)	1.640 (0.1258)	0.542 (0.0795)
Experiment 2			
Colour encoding			
* d*_*a*_	2.461 (0.1427)	2.207 (0.1093)	1.127 (0.0795)
* x*_*c*_	1.435 (0.1149)	1.228 (0.1035)	0.062 (0.0658)
Category encoding			
* d*_*a*_	1.860 (0.0981)	2.140 (0.1145)	1.311 (0.0790)
* x*_*c*_	0.908 (0.0910)	1.165 (0.1001)	0.259 (0.0659)

*d*_*a*_ represents memory sensitivity, *x*_*c*_ represents the placement of the middle response criterion location

### Two-high-threshold model analyses

**Fig. 3:**
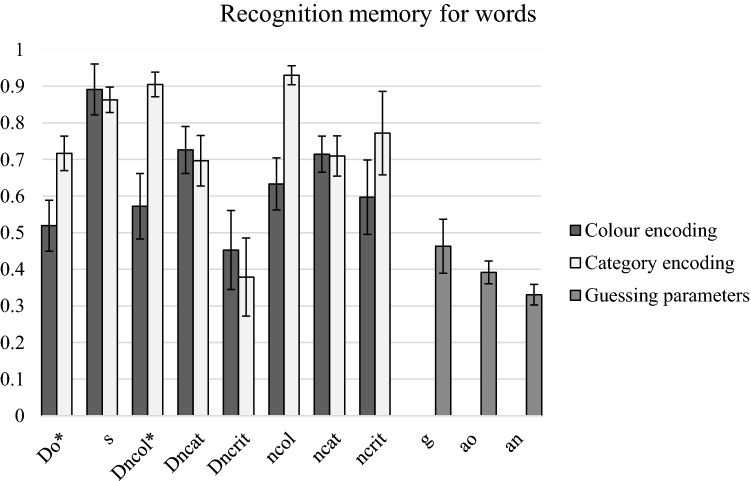
Bootstrapped estimates of the two-high-threshold model parameters and standard deviations obtained in Experiment 1. *D*_*o*_ = probability of old item detection, *s* = probability of high confidence “old” response, *D*_*n*_ = probability of new item detection, *n* = probability of high confidence “new” response, *g* = probability of guessing the status of an undetected item as old, *a*_*o*_ = probability of choosing with a high confidence an “old” response for an item guessed as old, *a*_*n*_ = probability of choosing with a high confidence a “new” response for an item guessed as “new”. Asterisks indicate significant differences between parameters for colour vs. category encoding conditions

Figure 3 shows bootstrapped estimates of 2HTM parameters and their standard deviations. The goodness of fit of the model to the empirical data was satisfactory, *G*^2^ (5) = 5.22, *p* = 0.39. The *D*_*o*_ detection of old words parameter was significantly higher in the category condition than in the colour encoding condition, Δ*G*^2^ (1) = 24.31, *p* < 0.001. In a similar way, the *D*_*ncol*_ detection parameter of the colour lures was significantly higher in the category condition than in the colour-encoding condition, Δ*G*^2^ (1) = 27.77, *p* < 0.001. Finally, the *n*_col_ parameter representing a high confidence of “new” response tended to be higher in the category condition than in the colour condition, Δ*G*^2^ (1) = 3.63, *p* = 0.06.

When comparing detection (*D*_*n*_) across the types of lures, an interesting crossover of effects can be observed between the encoding conditions. In the category condition, colour lures were significantly better detected than category lures, Δ*G*^2^ (1) = 13.26, *p* < 0.001, whereas in the colour condition, category lures were better detected than colour lures, Δ*G*^2^ (1) = 4.49, *p* < 0.04. Critical lures were the worst detected in almost all conditions in comparison with both colour and category lures, Δ*G*^2^ (1)s > 12.47, ps < 0.005, the only exception occurred in the colour condition, where the critical lures were not detected differently than the colour lures, Δ*G*^2^ (1) = 2.03.

In sum, Experiment 1 confirmed a typical positive effect of LoP for words, that is, the category encoding task resulted in a significant increase in hit rates and decrease in false alarms rates. Both SDT and 2HT analyses also confirmed the LoP effect. Turning to the effects on false memory for specific lures, the critical lures were significantly more often falsely accepted than the colour or the category lures. However, a predicted interaction effect was also observed—while for the category lures the level of false alarms was similar in the colour and the category encoding conditions, for the colour lures, the category encoding condition resulted in a salient drop in false alarms in comparison with the colour condition. This suggests that the participants effectively rejected the lures matching in colour but inconsistent in category with the targets only when they focused their attention on categories at study. It was confirmed by the SDT analysis of the response criterion placement that the participants were more conservative in accepting colour lures in the category condition than in the colour-encoding condition, indicating an influence of retrieval monitoring. Moreover, 2HT analyses indicated that colour lures were best detected as lures in the category encoding condition and, what is more, this was done with high confidence, again suggesting the recall-to-reject monitoring.

## Experiment 2

The aim of the second experiment was to demonstrate the processing-material interaction effects on false recognitions with pictorial materials. Differences in the nature of memory traces for visual and verbal materials are expected on the basis of, for example, the observation that the accuracy of visual recognition of object drawings is uncorrelated with the accuracy of recognition of the verbal labels of the same stimuli (Bahrick & Bahrick, 1971; Bahrick & Boucher, 1968). Moreover, the greater physical distinctiveness of pictures in comparison with words, suggested by the sensory-semantic model, may result in enhanced picture encoding during the perceptual orienting task (Intraub & Nicklos, 1985). As in Experiment 1, LoP was manipulated by colour or category naming orienting tasks, and lures differed according to the kind of consistent and differentiating features. In Experiment 1, our results suggested that colour lures inconsistent in category are most effectively rejected when participants attend to the category of studied words. In Experiment 2, we predicted that category lures inconsistent in colour are effectively rejected when participants attend to the colour of studied pictures.

### Methods

#### Participants

Seventy-one undergraduates volunteered to participate in the experiment in exchange for course credits. The mean age of the participants was 20.0 years (SD = 0.84); 13 were men. They were assigned to the colour naming (*N* = 35) or the category naming encoding conditions (*N* = 36). A sensitivity analysis using G*Power 3.1 (Faul et al., 2007), assuming a power of 0.80, indicated that our sample size (*N* = 71) allowed us to detect a small-to-medium effect size of *f* = 0.15. Baddeley and Hitch (2017) reported for their experiments that LoP effects for targets were modest across the visual stimuli. Their mean effect size *d* = 0.5, which corresponds to *f* = 0.25, was therefore larger than the minimum effect that can be detected in our experiment.

### Materials

The material comprised 63 full-colour photographs of door scenes, 58 of which were selected from a computerized database prepared by Baddeley et al. (2016), in which the door scenes are categorized along a set of various dimensions such as function, colour, age, condition, and shape, etc. (the database is available on the website: http://www.york.ac.uk/res/doors). The remaining five photos were taken from the Internet since we did not find enough stimuli in the database that unequivocally fulfilled our criteria. We selected the stimuli on the basis of the features from two dimensions: function and colour. The stimuli belonged to the following sets: (a) 45 targets or critical lures: 15 domestic doors, 15 church doors, and 15 garage doors; for each of these categories, a third were predominantly brown, a third were green, and a third were grey; (b) six category lures: doors belonging to the same categories as the targets but differing in their predominant colour (blue or red); (c) six colour lures: doors whose predominant colour was brown, green or grey (as targets) but they differed from the targets in the category (gate or hut doors); and (d) six primacy or recency buffers similar in category and colour to the targets.

#### Procedure

At study, the participants were instructed to try to memorize all the presented pictures and to answer the orienting question using a keyboard. In the category condition, the participants were asked to assess each door in terms of whether it looks like a domestic, church or garage door. In the colour condition, they were asked to indicate whether the predominant colour of the door is brown, green or grey.

At study, 36 target stimuli (3 colours × 3 categories × 4 exemplars) were presented in a random order at a rate of 6 s with an interstimulus interval of 250 ms. Three pictures were added as buffers at the beginning and another three at the end of the study list. All the stimuli were shown in the centre of the computer screen with a black background. The response options were prompted in a white frame below the door scene picture.

At test, 18 targets (3 colours × 3 categories × 2 exemplars) mixed with 9 critical lures (3 colours × 3 categories × 1 exemplar), 6 colour lures (3 colours × 2 exemplars), and 6 category lures (3 categories × 2 exemplars) were presented in a random order. The assignment of the stimuli to the targets vs. the critical lures was counterbalanced across the participants. As in Experiment 1, the participants were asked to recognize items using a 4-point confidence scale, at a self-paced rate.

## Results

At study, the participants correctly answered 97.4% and 96.0% of the orienting questions for the colour and the category conditions, respectively.

### Standard statistical analyses (ANOVA)

The mean proportions of targets and lures acceptances across the 4-point confidence scale are reported in the lower half of Table 1. For the hit rate as the dependent variable, one-way ANOVA indicated no difference between the category condition (*M* = 0.83, SD = 0.168) and the colour condition (*M* = 0.84, SD = 0.126), *F*(1) = 0.18.

For the false alarm rate a 3 (distractor type) × 2 (encoding task) mixed ANOVA was calculated, with the distractor type manipulated within-subjects, and the encoding task manipulated between subjects. Main effects of distractor type, *F*(1.82) = 110.35, *p* < 0.001, *η*_*p*_^2^ = 0.61, and an interaction effect, *F*(1.82) = 6.16, *p* = 0.004, *η*_*p*_^2^ = 0.08, were revealed; however, no effect of encoding condition was observed, *F*(1) = 0.06. Post hoc comparisons showed significantly more false alarms for critical lures (*M* = 0.44, SD = 0.219) than category lures (*M* = 0.13, SD = 0.229), *t*(70) = 10.57, *p*_Bonf_ < 0.001, *d* = 1.25, and colour lures (*M* = 0.12, SD = 0.231), *t*(70) = 12.67, *p*_Bonf_ < 0.001, *d* = 1.50, but there was no difference between category and colour lures, *t*(70) = 0.55. No significant differences were found in false alarm rate for all three kinds of lures depending on the encoding condition; however, for the category lures, false alarm rate tended to be higher in the category condition (*M* = 0.18, SD = 0.239) than in the colour condition (*M* = 0.08, SD = 0.211), *t*(69) = 1.77, *p* = 0.08, *d* = 0.42.

Parallel results of the analysis of mean confidence ratings for targets and lures on the 4-point confidence scale are reported in “Appendix 1”.

### Signal detection analyses

Parameter estimates of the SDT model are presented in Table 2. The memory sensitivity parameter *d*_*a*_ was significantly better in the colour condition than in the category condition, *z* = 3.47, *p* < 0.001, but only when the category lures were used for the false alarm rates. Other comparisons, that is, when the colour lures or the critical lures were used, showed no differences between the encoding conditions.

The placement of the middle response criterion *x*_*c*_ in the case of the category lures was significantly more conservative in the colour condition than in the category condition, *z* = 3.60, *p* < 0.001. Conversely, for the critical lures, a more liberal criterion placement was observed in the colour condition than in the category condition, *z* = 2.11, *p* < 0.04. No difference in criterion placement was observed for the colour lures.

### Two high-threshold model analyses

**Fig. 4:**
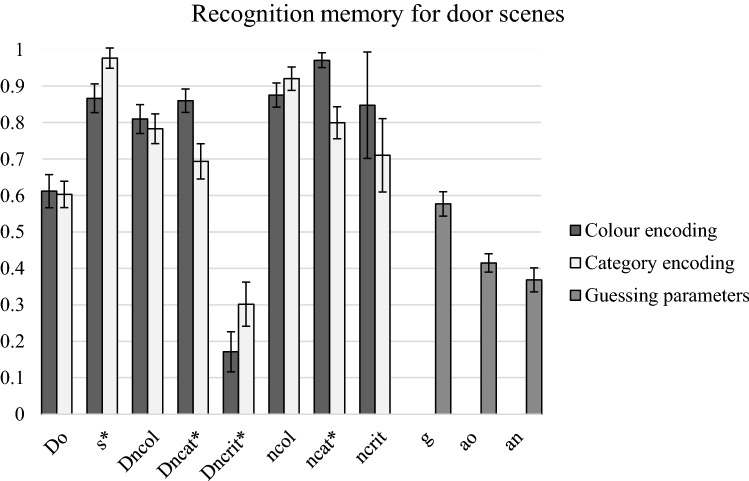
Bootstrapped estimates of the two-high-threshold model parameters and their standard deviations obtained in Experiment 2. *D*_*o*_ = probability of old item detection, *s* = probability of high confidence “old” response, *D*_*n*_ = probability of new item detection, *n* = probability of high confidence “new” response, *g* = probability of guessing the status of an undetected item as old, *a*_*o*_ = probability of choosing with a high confidence an “old” response for an item guessed as old; *a*_*n*_ = probability of choosing with a high confidence a “new” response for an item guessed as “new”. Asterisks indicate significant differences between parameters for colour vs. category encoding conditions

Figure 4 shows bootstrapped estimates of 2HTM parameters and their standard deviations. The goodness of fit of the model to the empirical data was highly satisfactory, *G*^2^ (5) = 3.974, *p* = 0.55. In this experiment, the detection of old items (*D*_*o*_) was not different between the encoding conditions, Δ*G*^2^ (1) = 0.05; however, the *s* parameter representing the probability of “definitely yes” responses to the detected items was significantly higher in the category condition, Δ*G*^2^ (1) = 4.33, *p* = 0.04. The *D*_ncat_ detection parameter of the category lures was significantly higher in the colour condition than in the category condition, Δ*G*^2^ (1) = 8.75, *p* = 0.003. In contrast, the detection of critical lures *D*_ncrit_ was significantly better in the category condition than in the colour condition, Δ*G*^2^ (1) = 3.96, *p* < 0.05. Finally, the *n*_cat_ parameter was significantly lower in the category than in the colour condition, Δ*G*^2^ (1) = 9.49, *p* = 0.002. When detection parameters were compared across the lure types, the critical lures were significantly worse detected in both encoding conditions than the category lures and the colour lures, Δ*G*^2^ (1)s > 32.20, ps < 0.001.

In sum, Experiment 2 showed neither positive nor reversed LoP effects for the door scenes, both in terms of hit rate and response confidence level. However, 2HTM analyses indicated that the targets detected as old were more often accepted with high confidence in the category condition than in the colour encoding condition. As in Experiment 1, the false alarm rates for the critical lures were significantly higher than for the colour or the category lures. An interaction effect was also observed—while for the colour lures the level of false alarms was similar in the colour and the category encoding conditions, for the category lures, the colour condition resulted in a decrease in the false alarm rate and confidence rating in comparison with the category encoding condition. The response criterion placement indicated that the participants were more conservative in accepting the category lures in the colour condition than in the category condition. This suggests that the participants effectively rejected the category lures with inconsistent colour only when they focused on the colours of the targets at study. Moreover, 2HTM analyses showed that the category lures were best detected as lures in the colour encoding condition and, what is more, this was done with a higher confidence.

### Cross-experiment comparisons

To examine the role of material on monitoring for inconsistent features depending on the encoding orientation, we compared results between Experiments 1 and 2. We focused solely on false alarm rates for category and colour lures and interaction effects with material type. A 2 (lure type: category, colour lure) × 2 (encoding task: category, colour) × 2 (material type: pictorial, verbal) mixed ANOVA showed two interaction effects: between lure type and encoding task, *F* (1) = 14.47, *p* < 0.001, *η*_*p*_^2^ = 0.11, and between material type and encoding task, *F* (1) = 3.98, *p* < 0.05, *η*_*p*_^2^ = 0.03. This indicates that colour vs. category lures recognition depends on the encoding task, and that lures are more often recognised as new in the case of pictorial material but only in the colour encoding condition.

In order to test interaction effects in the multinomial processing tree model we used a method recently recommended by Kuhlmann et al. (2019). This method defines interactions as in Log-Linear Models, that is, the invariance of parameter ratios across the cells is tested rather than an invariance of parameter differences. For data obtained in Experiments 1 and 2, we created a combined model which goodness of fit was highly satisfactory, *G*^2^ (10) = 9.19, *p* = 0.51. From this model, we derived a reparametrized model using a method implemented in the *multiTree* computer program (Moshagen, 2010). The reparametrization method allows replacing one of the model parameters by a novel parameter that reflects decreases in the original parameter (here, this is a shrinkage factor for the detection parameter or the high confidence response parameter in the colour condition compared to the category condition). The shrinkage parameters can be than compared across conditions (here, material type) to assess whether an interaction is present or not (for more details see: Kuhlmann et al., 2019).

We focused on testing interactions between study condition (colour encoding, category encoding) and material type (pictorial, verbal) for detection of colour lures and category lures and parameters representing high confidence “new” responses to these lures. For the *D*_ncat_ detection parameter of the category lures, no interaction was found, Δ*G*^2^ (1) = 0.90. However, the interaction effect was significant for the *D*_ncol_ detection parameter of the colour lures, Δ*G*^2^ (1) = 13.12, *p* < 0.001, which is a simple dissociation since there was a main effect of encoding condition for words but not for pictures. For the *n*_cat_ parameter and the *n*_col_ parameter representing a high confidence of “new” responses, the interaction effects were not significant. Moreover, analysis revealed the interaction effect for the *D*_*o*_ parameter, Δ*G*^2^ (1) = 4.84, *p* < 0.03, which is a simple dissociation since there was a main effect of encoding condition for words but not for pictures. No interaction was found for the *s* parameter. The most important conclusion from these comparisons across encoding conditions and experiments is that the detection of colour lures (which are inconsistent in category) interacts with the material type being the best in the category condition for words but not for pictures.

## General discussion

Following the research of Baddeley and Hitch (2017) on LoP effects on pictorial vs. verbal stimuli, we studied the encoding orientation effects on false memory for the lures that matched the perceptual or the semantic features with the target stimuli. We built our predictions on the literature concerning the recall-to-reject and the retrieval monitoring processes (e.g., Chan et al., 2005; Gallo, 2004; Gallo et al., 2008; Jacoby et al., 2005; Rugg & Wilding, 2000). We assumed that our participants would attempt to recapitulate the category or the colour orienting tasks when assessing the test probes. In consequence, they will be able to detect a mismatch of colour or category and reject the mismatching lure items. We expected that such a recall-to-reject process is particularly effective for deeply encoded words due to their enhanced distinctiveness (Gallo et al., 2008). However, the predictions for the pictures were not so straightforward, since there is rationale for semantic (Baddeley & Hitch, 2017) as well as pictorial processing (Nieznański, 2020) to be beneficial for picture distinctiveness at retrieval. Therefore, the main contribution and novelty of this research regards the results for pictorial materials.

Our results clearly supported the effects of retrieval orientation on disqualifying monitoring for both words and pictures. We demonstrated that the participants successfully rejected the colour lures that mismatched the category with the target words and, in mirror symmetry, they successfully rejected the category lures that mismatched the colour with the target pictures. For example, in the case of a blue category lure, the participants recalled that the studied pictures were brown, green or grey, hence, they excluded the blue test probe. The probability of using such a strategy is greater when they classified the targets at study according to their colours. Complementarily, in the case of a colour lure being an exemplar of furniture, the participants excluded this lure since they remembered that they studied exemplars of animals, clothes, and fruits but not of furniture. And again, such an error-editing process is enhanced when the words were semantically processed at encoding. What is important is that we found these interaction effects on false memories when significant LoP effects on accurate memories were observed (Experiment 1) as well as when such effects were not present (Experiment 2). This suggests that effects on false memories are not the simple function of the effectiveness of attended features encoding. Experiment 2 suggest that the recapitulation of the orienting task at retrieval affects rejection of lure items inconsistent in color (i.e., detection of category lures as new items), even if the color orientating task does not affect memory for targets (i.e., detection of old items). The lack of LoP effects for door scenes comes across as inconsistent with the observation of Baddeley and Hitch (2017) of modest positive LoP effects for pictures, however, note that they used distractors equivalent to our critical lures, limiting the distinctiveness of the distractors in comparison with our experiments.

In both experiments, we showed that critical lures matching the targets both in colour and category are most often falsely accepted. On the one hand, critical lures are test probes that evoke the highest global-matching with encoded memories. In our experiments, this manifested, for example, in saliently lower values of the *D*_ncrit_ distractor-detection parameter estimates for the critical lures in comparison with other types of lures in both experiments and in almost all the encoding conditions. On the other hand, for critical lures, correct rejection cannot be supported by a simple disqualification due to mismatching the colour or the category. And in fact, in our results, the placement of the *x*_*c*_ response criterion was evidently most liberal for the critical lures across all conditions, suggesting less effective retrieval monitoring for the critical lures (cf. Huff & Bonder, 2013). However, the *n* parameter of 2HTM, representing the probability of high confidence “new” responses, was only numerically lower for the critical lures than for other types of lures, indicating that the frequency of using the recall-to-reject strategy is not significantly different across the lure types (cf. Rotello et al., 2000).

An alternative interpretation of our results can be considered, namely that the false recognitions were solely due to error-inflating encoding processes without any influence of the disqualifying monitoring processes. We might assume that lures differ in their effectiveness of inducing false memories—probes matching in colour (or category) might be stronger or weaker lures depending on the kind of features enhanced by the encoding task. Hence, category encoding, relative to colour encoding, should result in a higher level of false recognition of category lures and, cognately, colour encoding should result in a higher level of false acceptances of colour lures. This pattern of effects should be in the same direction, though probably of different strength, for words and pictures. Such a scenario was partially supported by higher false alarms for colour lures in the colour condition for words, and higher false alarms for category lures in the category condition for pictures. However, this was opposed by the lack of the two remaining symmetrical effects in that it cannot explain why semantic encoding for words did not result in high false alarms for category lures in comparison with the colour encoding condition, and why colour encoding, relative to category encoding, did not increase false alarms for colour lures for pictures. Moreover, our SDT analyses demonstrated effects on both memory sensitivity and the response criterion parameters, which confirms the contribution of both encoding and retrieval processes (Huff & Bonder, 2013). Therefore, we suggest, as in most contemporary theories of false memory (Arndt & Gould, 2006), that both error-inflating encoding processes and error-editing retrieval processes are needed to fully explain our interaction effects on false memories.

Most experimental research on false memory has been conducted using the well-known Deese/Roediger–McDermott (DRM) paradigm (Deese, 1959; Roediger & McDermott, 1995). In this task, participants study lists of words (e.g., *bed*, *rest*, *awake*, *dream…*) semantically associated with a non-studied word (*sleep*), which is used as a critical lure during a memory test. High false acceptances of critical lures in the DRM paradigm are explained as resulting from the similarity of gist traces between the list-items and the critical lure (e.g., Brainerd & Reyna, 2002; Nieznański et al., 2019) or from spreading activation from the studied list-words to the non-studied critical lures (e.g., Roediger et al., 2001). A critical lure can be rejected at test if the participant effectively monitors the origin of the feeling of familiarity evoked by this lure (e.g., Bruce et al., 2004; Carmichael & Gutchess, 2016; Nieznański et al., 2018). Reductions in false memories of critical lures are achieved when participants are warned about the memory illusion before they study the DRM lists. They probably employ strategic processes to identify potential critical words at study to subsequently reject them at test, and the effectiveness of this control process is probably dependent on working memory capacity (e.g., Neuschatz et al., 2003; Nieznański & Obidziński, 2019; Tkaczyk & Nieznański, 2013). However, even when subjects are not warned before the study, a critical lure may be identified as the missing theme or gist of the study list and used by the recall-to-reject process—as suggested by the observation of Carneiro et al., (2009; cf. Carneiro et al., 2012, 2014), that lists for which the gist/theme can easily be identified produce lower levels of lure acceptances. In our study, it seems that the recalled colours and categories played a similar role to the identified themes of lists in the studies of Carneiro and colleagues (cf. Nieznański & Tkaczyk, 2017). Our results showing processing-material interactions correspond with Chan et al. (2005) observations made with semantically vs. phonologically associated lists of words. They showed that the meaning orienting task, in comparison with the sound orienting task, leads to better target memory and higher probability of false recognition for semantically associated lists. However, for phonologically associated lists, it is the sound orienting task that leads to better target memory and higher probability of false recognition. In our research we extended such observations of processing-material interactions to the pictorial material and to the manipulation of features that differentiate distractors.

In conclusion, we demonstrated that encoding orientation affects error-editing processes differently, depending on the to-be-remembered materials. We assumed that participants strategically or spontaneously recapitulate the encoding orientation at test, which influences the information they use to make recognition judgments (e.g., Jacoby et al., 2005). For pictures, inconsistent perceptual features are effectively rejected when perceptual processing is mentally recreated at test. For words, inconsistent semantic features are successfully monitored when categorical processing is reimplemented at retrieval.

## Data Availability

Raw data are available at https://osf.io/st6rc/.

## Appendix 1: Standard statistical analyses of confidence ratings in Experiments 1 and 2

For Experiment 1, the mean ratings for the targets and the three types of lures on the 4-point confidence scale are reported in the upper half of Table 1. Confidence ratings for targets were significantly higher in the category than in the colour encoding condition, *F*(1) = 8.39, *p* < 0.01, *η*_*p*_^2^ = 0.14. Confidence ratings for lures were subjected to a 3 (distractor type) × 2 (encoding task) mixed ANOVA, which revealed a main effect of distractor type, *F*(2) = 30.51, *p* < 0.001, *η*_*p*_^2^ = 0.37, a main effect of encoding task, *F*(1) = 5.27, *p* < 0.05, η_p_^2^ = 0.09, and an interaction effect, *F*(2) = 15.91, *p* < 0.001, *η*_*p*_^2^ = 0.24. Post hoc comparisons showed that participants gave higher confidence ratings to the critical lures than the category lures, *t*(52) = 6.73, *p*_Bonf_ < 0.001, *d* = 0.92, and colour lures, *t*(52) = 5.90, *p*_Bonf_ < 0.001, *d* = 0.81; however, the difference between category and colour lures was not significant, *t*(52) = 1.14. Encoding task conditions resulted in very similar levels of mean ratings for the category lures and for the critical lures, however, for the colour lures, the ratings were significantly higher in the colour than in the category encoding condition, *t*(44.26) = 6.03, *p* < 0.001, *d* = 1.65.

The mean ratings for the targets and the three types of lures on the 4-point confidence scale obtained in Experiment 2 are reported in the lower half of Table 1. Confidence ratings for targets did not differ depending on the encoding condition, *F*(1) = 0.29, *η*_*p*_^2^ = 0.004. Confidence ratings for lures were subjected to a 3 (distractor type) × 2 (encoding task) mixed ANOVA, which revealed a main effect of distractor type, *F*(2) = 140.33, *p* < 0.001, *η*_*p*_^2^ = 0.67, and an interaction effect, *F*(2) = 9.91, *p* < 0.001, *η*_*p*_^2^ = 0.13. There was no effect of encoding task, *F*(1) = 0.76, *η*_*p*_^2^ = 0.01. Post hoc comparisons showed that participants gave higher confidence ratings to the critical lures than the category lures, *t*(70) = 11.61, *p*_Bonf_ < 0.001, *d* = 1.38, and colour lures, *t*(70) = 15.03, *p*_Bonf_ < 0.001, *d* = 1.78, the difference between category and colour lures was not significant, *t*(70) = 0.71. Encoding task conditions resulted in very similar levels of mean ratings for the colour lures and for the critical lures; however, for the category lures, the ratings were significantly lower in the colour than in the category encoding condition, *t*(69) = 2.92, *p* = 0.005, *d* = 0.69.

## Footnotes

1 Items with incorrect responses to orienting questions were not excluded from the analyses since we assumed that errors were random slips in reaction execution rather than deficits of participants’ knowledge or perception that could last also during memory test.

## Code availability

Multinomial model equations are available at https://osf.io/st6rc/.
